# Human identification: an investigation of 3D models of paranasal sinuses to establish a biological profile on a modern UK population

**DOI:** 10.1007/s00414-024-03179-2

**Published:** 2024-02-13

**Authors:** Madeline Robles, Sherry Nakhaeizadeh, Carolyn Rando, Ruth M. Morgan

**Affiliations:** 1 UCL Department of Security and Crime Science, 35 Tavistock Square, London, WC1H 9EZ UK; 2 UCL Centre for the Forensic Sciences, 35 Tavistock Square, London, WC1H 9EZ UK; 3 School of Applied Sciences, College of Health, Science and Society, University of the West of England, Coldharbour Lane, Bristol, BS16 1QY UK; 4 UCL Institute of Archaeology, 31-34 Gordon Square, London, WC1H 0PY UK

**Keywords:** Forensic science, Forensic anthropology, 3D modelling, Computed tomography, Paranasal sinuses

## Abstract

Medical imaging is a valuable source for facilitating empirical research and provides an accessible gateway for developing novel forensic anthropological methods for analysis including 3D modelling. This is especially critical for the United Kingdom (UK), where methods developed from modern UK populations do not currently exist. This study introduces a new approach to assist in human identification using 3D models of the paranasal sinuses. The models were produced from a database of 500 modern CT scans provided by University College London Hospital. Linear measurements and elliptic Fourier coefficients taken from 1500 three-dimensional models across six ethnic groups assessed by one-way ANOVA and discriminant function analysis showed a range of classification rates with certain rates reaching 75–85.7% (*p* < 0.05) in correctly classifying age and sex according to size and shape. The findings offer insights into the potential for employing paranasal sinuses as an attribute for establishing the identification of unknown human remains in future crime reconstructions.

**Supplementary Information:**

The online version contains supplementary material available at 10.1007/s00414-024-03179-2.

## Introduction

Technological advancements in medical imaging are facilitating and validating empirical research to underpin new methods in forensic anthropology [1, 2]. These approaches offer advantages not only in individual cases but also in facilitating empirical research that allows for validation or improvements over existing methods [3, 4]. In certain cases, these advancements may also introduce novel methods of analysis into the discipline [5–7]. Although radiographic imaging is commonplace, the manner in which these images can be obtained, processed and visualised is constantly advancing [8]. The accuracy and reliability of 3D imaging to depict skeletal elements has been well established [9–11], and using computer software programmes for visualisation allows for incredibly powerful analysis [5, 12, 13]. These new developments in data visualisation are at the peak of technological innovation developed for medical and forensic science applications [4, 5, 14–16].

Medical imaging such as computed tomography (CT) and magnetic resonance imaging (MRI) are becoming more widely used across forensic domains including post-mortem examination and disaster victim identification (DVI) in mass death scenarios [5, 17]. These advancements offer new areas for research in examining anatomical structures and create opportunities for quicker data collection and creating larger datasets [2]. The ability to produce three-dimensional reconstructions of an original scanned object allows for improved accuracy and precision when applying traditional anthropometric and osteometric methods for analysis [5, 7, 11, 18, 19]. Exercising imaging technology in DVI situations, in particular, boasts several benefits which include isolating discrete individuals prior to DNA sampling or anthropological analysis in cases of comingled remains [5]. Furthermore, it allows a larger number of physically removed practitioners to assist with examination of the remains which is crucial for providing rapid identifications [2]. Mobile computed tomography is growing in its application in DVI and was utilised in the 2017 Grenfell tower fires that occurred in London, UK [20]. In this scenario, a mobile CT scanner was used to produce 3D reconstructions of entire skeletal remains and areas for potentially identifiable features including medical implants and internal sexual organs to aid identification. Many different anatomical features have been demonstrated to offer valuable insight in these contexts, and Boer et al. [2] state that the anatomical traits including the morphology of the paranasal sinuses may also provide useful information in DVI situations [21, 22]

The use of CT scans and digital imaging also means that locating and accessing documented human skeletal collections is no longer a requirement for developing or assessing identification methods [2, 5]. The use of medical imaging and utilising these modes of visual analysis is vital, especially when access to skeletal collections is not possible due to cultural, financial or travel restrictions [23], especially within Europe, where contemporary skeletal collections do not currently exist [8, 23]. Criticism for the use of medical imaging databases within forensic anthropology is mainly predicated on misreading CT images and mistaking trauma for normal variations which could result in erroneous identifications or lower accuracy rates compared to real bone [19, 24, 25]. A study by Bertolglio et al. [24] investigated crania CT models for non-metric analysis and found that the models were good representations overall but identified problematic areas of missing bone or anatomic details. Bertolglio et al. [24] therefore suggested that as software advances, so will the reconstructions. However, a more proactive solution is for forensic anthropologists to receive training in medical imaging and 3D model analysis to ensure accurate reproductions and avoid misinterpretation at the first instance [26–28]. This also highlights the need for more studies addressing the application of metric and non-metric forensic anthropology methods on 3D models across different visualisation platforms to provide and demonstrate that there is an empirical foundation across platforms.

Employing medical imaging databases for research offers access to modern populations for method development and therefore does not have ties to colonisation similar to many current skeletal collections [23, 29]. Furthermore, the accuracy of skeletal collections in representing contemporary and even past populations has been called into question [19, 23, 30]. With the increased availability of digital imaging databases, researchers are able to access and undertake research with greater ease than before and, in some cases, at much lower financial cost. For example, the Radiology-Pathology Centre for Forensic Imaging (CFI) at the University of New Mexico (https://nmdid.unm.edu/) recently made 15,000 whole body-decedent CT scans available for research (upon request). The Smithsonian Institute also has a digital collection as well as the IMPACT Radiological Mummy Database, to name a few [31]. Furthermore, forensic institutes routinely perform CT scanning prior to autopsy which constantly increases the datasets of modern populations potentially suitable for research [2, 31]. This ease of access to this type of data will allow complementary studies across a diverse range of populations. This is important for the forensic anthropology domain, where the majority of traditional approaches were developed using North American populations and yet are used globally to identify unknown human remains [32–35]. Furthermore, confirmation studies have shown conflicting accuracy rates when traditional methods were applied to skeletal remains outside of the USA. Complementary research that reflects modern UK populations is ultimately needed to support new methods of measurement and analysis to provide valid and robust identifications within the UK.

The study presented here builds on the research presented by Robles et al*.* [7] which presents a new approach for establishing a biological profile using 3D models of the paranasal sinuses from a modern UK population. Linear measurements and elliptic Fourier coefficients taken from 1500 three-dimensional paranasal sinus models across six ethnic groups were assessed by one-way ANOVA and discriminant function analysis to determine their accuracy in predicting age, sex and ethnicity. The results showed a range of classification rates with some rates reaching 75–85.7% (*p* < 0.05) in correctly classifying age and sex according to size and shape. The results presented in this study show the potential of employing 3D imaging methods to estimate the age and sex of modern unknown individuals using the paranasal sinuses.

## Method

This study employed the method presented by Robles et al*.* [6] to produce 3D models of CT scans using *3D Slicer*™. The method outlined here was devised to determine if the size and shape of the paranasal sinuses could establish age, sex and ethnicity, which could then assist with human identification in crime reconstructions. The primary difficulty that has become the focus in recent years is how to address or define ancestry and whether this is even appropriate. Many publications have used the terms ‘race’, ‘ancestry’ and ‘ethnicity’ inconsistently and without definition [36, 37]*.* A number of authors, particularly within forensic anthropology, have offered definitions that outline various aspects of an individual’s identity and have introduced additional terms such as ‘population affinity’ and ‘bioaffinity’ [38, 39]. Therefore, it is important to note that the known age, sex and ethnic groups within this study were self-identified by the patient and categorised by the NHS under the term ‘ethnicity’. To maintain consistency and transparency, this study, therefore, had to adopt the term ‘ethnicity’ as this had been self-identified by the patients. Although the capacity for forensic discrimination of certain sinuses exists [40–45], their complex anatomical structure has represented significant challenges for being able to produce standardised methods of measurement across each paranasal sinus [6, 7, 46]. The intention of creating 3D models was to develop an empirical measure to establish an evidence base that can provide transparency in the final inferences and analysis made by the expert in crime reconstruction.

### CT acquisition

For this study, 500 clinical sinus CT scans from University College London Hospital (UCLH) were acquired. The CT scans were anonymised and randomised by the picture archiving and communications (PACS) department prior to receipt, with only self-reported age, sex and ethnic group provided for this study. The CT scans were provided in DICOM (Digital Imaging and Communications in Medicine) format using 100 kVp at 1-mm slice thickness. In the hospital, these CT scans were acquired using a variety of CT scanners with varying imaging conditions and parameters. Variations in imaging conditions in producing three-dimensional models are inevitable as they depend on the platforms and scanners available [47]. However, studies have shown that these variations are minor and still sufficiently precise where the point-to-point distance variation is within the accepted accuracy level of ± 2 mm [47, 48]. Consequently, the scans included in this database were derived from six different scanners:

- GE Medical Systems HiSpeed
- SIEMENS Sensation 16
- SIEMENS Sensation 64
- SIEMENS SOMATOM Definition AS + 
- TOSHIBA Aquilion ONE
- TOSHIBA Aquilion Prime

### Data collection

The CT scans were reviewed prior to data collection to identify which scans needed to be excluded from the analysis. CT scans were excluded from analysis if the quality of the scans was distorted or did not provide clear images of the sinuses. The sample included individuals with missing frontal sinuses or missing a maxillary sinus. In these cases, the other sinuses present and visible were included in the analysis. The initial sample size prior to the statistical analyses is displayed in Table 1.

**Table 1:** Sample breakdown

	Male	Female	Age group			Total
			20–39 years	40–59 years	60 +	
*Frontal sinus*						
* White British*	21	27	12	21	15	48
* Black African*	22	21	15	19	9	43
* Chinese*	8	15	5	8	10	23
* Black Caribbean*	27	28	22	26	8	56
* Indian*	35	37	28	24	20	72
* Pakistani*	27	28	27	25	3	55
*Total*						**297**
*Max L sinus*						
* White British*	22	26	12	19	17	48
* Black African*	20	24	15	20	9	44
* Chinese*	8	18	5	11	10	26
* Black Caribbean*	27	26	21	26	6	53
* Indian*	30	37	25	24	18	67
* Pakistani*	26	28	27	25	2	54
*Total*						**292**
*Max R sinus*						
* White British*	22	26	11	21	16	48
* Black African*	23	21	15	19	10	44
* Chinese*	6	17	5	11	7	23
* Black Caribbean*	29	28	22	28	7	57
* Indian*	31	41	30	24	18	72
* Pakistani*	27	27	27	23	4	54
*Total*						**298**
*Ethmoid sinus*						
* White British*	17	28	13	18	14	45
* Black African*	22	25	17	20	10	47
* Chinese*	9	16	5	11	9	25
* Black Caribbean*	28	31	22	28	9	59
* Indian*	26	37	27	20	16	63
* Pakistani*	27	31	30	24	4	58
*Total*						**297**
*Sphenoid sinus*						
* White British*	23	28	12	23	15	50
* Black African*	18	24	16	18	8	42
* Chinese*	8	19	5	12	10	27
* Black Caribbean*	30	33	23	30	10	63
* Indian*	34	39	29	26	18	73
* Pakistani*	30	31	30	27	4	61
*Total*						**316**
*Overall total*	**1500**					

### 3D model production

Producing 3D models of the paranasal sinuses was integral for collecting accurate data, given that sinuses are 3D entities [7, 18, 49]. Therefore, this study employed *3D Slicer*™ [50] following the method designed by Robles et al*.* [6] from cropping the volume, establishing the thresholding parameters and segmenting to the final post-processing in *Meshlab*™ [51] to produce individual 3D Stereolithic (STL) models that allowed for easy export/import to other platforms for further processing or analysis.

### Linear measurements

To determine if there was a statistically significant relationship between the size of the paranasal sinuses and age, sex and ethnic group, linear and volumetric measurements were collected from the 3D models. The data collection process follows the step-by-step procedure presented by Robles et al*.* [7]*.* The measurements taken were computer-assisted and not completed manually. This allowed for a more precise, consistent and rapid analysis process. The 3D models were imported to *Meshlab™* [51] which then provided the dimensions of the structure in millimetres by selecting the ‘*Quality Measure and Computations*’ and ‘*compute Geometric Morphometrics*’ filter wherein the dimensions of height (*y*-axis), length (*x*-axis), width (*z*-axis) and volume were computed and displayed [7].

### Shape coordinates

To determine if the shape of the paranasal sinuses showed a statistically significant relationship to age, sex and ethnic group, *x*,*y* coordinates were collected from the 3D models. This study employed the same method presented by Robles et al. [7] using *ImageJ* ™ and *Past3*™ [52] to collect and analyse Cartesian coordinates from 3D models of the paranasal sinuses. The digital outline summarises the shape of an open or closed curve without fixed landmarks [53]. This method applied Elliptic Fourier Analysis and, therefore, does not require a specific starting point or homologous-space points, which makes this approach conceivable to employ on the paranasal sinuses due to the extreme variation found between individuals [6, 54]. The *x*,*y* coordinates provided the initial data and were further analysed using elliptical Fourier analysis (EFA) [53]. To collect *x*,*y* coordinates, the 3D models were turned into 2D images and were transferred to *ImageJ* [7]. *ImageJ* was selected for this study because it was free and allowed for easy collection of *x*,*y* coordinates. The outline coordinates were extracted from each 3D model (see Robles et al. [7]) across each sinus (frontal, maxillary, ethmoid and sphenoid).

**Fig. 1:**
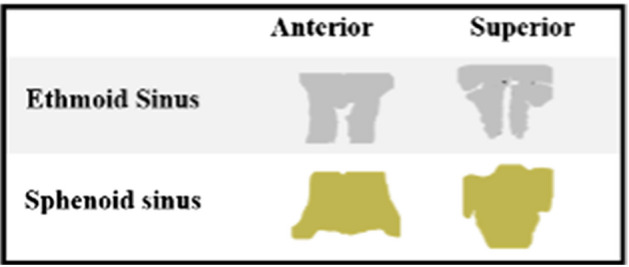
2D image of the ethmoid and sphenoid sinus used for outline analysis of anterior and superior orientation

However, two sets of *x*,*y* coordinates were taken from the ethmoid and sphenoid models in two different orientations. This was because outline analysis had not been completed on the ethmoid and sphenoid sinuses prior to this study. Consequently, there was no way of estimating which orientation of these sinuses would provide the maximum amount of morphological changes that might assist with providing age, sex and ethnicity indications. Therefore, the ethmoid and sphenoid models were orientated anteriorly and superiorly in *Meshlab™* [51] and were turned into 2D images (see Fig. 1). The subsequent *x*,*y* coordinates were taken from these 2D images. The coefficients taken from these structures were referred to as ‘anterior’ or ‘superior’ coefficients. Finally, in cases where there was an absent sinus (frontal or maxillary), the coordinates of the other sinuses were still recorded.

The final step of this process required the coordinates to be turned into elliptic Fourier coefficients. The coefficients were important as they allowed for discriminant analysis to be tested on these variables [7, 12, 55–57]. This also enabled a direct comparison between the linear and EFA coefficient classification results for accurately predicting age, sex and ethnic group [7]. The coefficients were calculated in the statistical platform *Past3*™ [52] and were provided in sets of four, where each set is defined as one *harmonic.* The calculation of the coefficients taken in *Past3*™ [52] ensured that the coefficients were invariant to size, rotation and starting point [52].

### Statistical analysis

The statistical software package IBM SPSS Statistics for Macintosh Software Version 26.0 (Armonk, NY: IBM Corp.) was used to carry out one-way ANOVA (analysis of variance) and discriminant function analysis (DFA). This was to determine how well the measurements acted as predictors in correctly classifying the original age, sex and ethnicity of the predetermined groups [7, 58]. DFA is an established approach for estimating if sex, age and ethnicity can be correctly classified using a particular method [7, 45, 59–62]. The result from this analysis is the ‘resubstitution estimate’, and this evaluates how well the discriminant function of the variables performs in their predictions by providing a classification rate [62].

First, descriptive statistics from each paranasal sinus measurement were analysed (at a 95% confidence interval), and results are available in Online resource 1. Prior to employing DFA, it was important to first understand if there were statistically significant mean group differences of the linear and EFA coefficients according to age, sex and ethnic group by running a one-way ANOVA. When statistically significant group differences were identified, DFA was carried out on the linear and EFA coefficients with 95% confidence intervals and with bootstrapping (1000 bootstrapped samples). The step-wise discriminant function was a useful parameter in estimating which variables contributed most to the discriminant rule and estimating those classification rates [63, 64]. This process eliminated the other variables that did not contribute and only provided the classification rate of the final statistically significant variables. Finally, all of the results from the discriminate function analyses were cross-validated with leave-one-out classification.

To address sex estimation, the linear measurements for each paranasal sinus were analysed with the known sex. The known sex was dummy coded (male = 1, female = 2) and tested against the recorded linear measurements. Age groups were analysed using DFA and were dummy coded from 1 to 3. The age groups were selected as allowed for by the distribution of the sample size and to fit the assumptions required for DFA [7, 58, 65] (see Table 1 for detailed dataset composition) and were 20–39 years (1), 40–59 years (2) and 60 + (3). Ethnicity estimation: the ethnic groups recorded in the original dataset were also dummy coded to allow for statistical analysis: White British (1), Indian (4), Pakistani (6), Chinese (2), Black African (3), Black and Caribbean (5).

## Results

In a similar manner to the preliminary study, the linear measurements and shape coefficients were extracted from the models and tested to determine if size or shape showed patterns across age, sex or ethnic group as well as identifying statistically significant classification percentages. The most notable results are presented here, with full results in Online resource 1.

### Linear measurement results: ethnic groups – key results

#### Chinese dataset (right maxillary sinus)

One-way ANOVA showed statistically significant group differences according to age (see Online resource 1). When the variables were entered into DFA together, the model did not fit the data (*p* = 0.141, *p* > 0.05). When all of the variables were entered into a step-wise classification (*F* to enter = 3.84, *F* to remove = 2.71), the classification rate of 61.9% (cross-validated at 57.1%) showed volume was the most discriminant and only variable in the analysis (*p* = 0.0.019, *p* < 0.05). One-way ANOVA did show statistically significant group differences according to sex (*p* < 0.05) (see supplementary table in Online resource 1). When the variables were entered into DFA together, the model did not fit the data (*p* = 0.188, *p* > 0.05). When all of the variables were entered into a step-wise classification (*F* to enter = 3.84, *F* to remove = 2.71), the classification rate of 85.7% (cross-validated at 85.7%) was statistically significant (*p* = 0.021, *p* < 0.05), which showed that width was the most discriminant variable (see Table 2).

**Table 2:** Summary of cross-validated classification rates and Wilks’ Lambda Statistics according to sex

	Wilks’ Lambda *(test of function 1)*	Sig	Classification (%)
Chinese dataset			
Right maxillary	0.751	0.021	85.7
Indian dataset			
Right maxillary	0.665	0	69.6

#### Indian dataset (right maxillary sinus)

One-way ANOVA did not show statistically significant group differences according to age (*p* > 0.05); therefore, DFA was not carried out (see Online resource 1). One-way ANOVA did show statistically significant group differences according to sex (*p* < 0.05) (see supplementary table in Online resource 1). When these variables were entered together into DFA, the classification rate of 80.4% (cross-validated at 69.6%) showed that the model was a good fit for the data (*p* = 0.000, *p* < 0.05) (see Table 2).

### EFA coefficient results: ethnic group

One-way ANOVA and discriminant function analyses (with 1000 bootstrapped samples) at 95% CI from the EFA coefficients (variables) were undertaken for each ethnic group. These results aimed to determine if there was a statistically significant relationship between the shape of the paranasal sinuses from each ethnic group to age, sex, and ethnicity that could assist with establishing a biological profile (see Table 3 and 4).

**Table 3:** Summary of Wilks’ Lambda Statistics for EFA and combination measurements

	Wilks’ Lambda*(test of function 1)*	Sig
Chinese dataset		
Frontal	0.706	0.02
Sphenoid (anterior)	0.699	0.006
Sphenoid (superior)	0.445	0
Black African dataset		
Ethmoid (anterior)	0.861	0.001
Ethmoid (anterior)	0.81	0.001
Indian dataset		
Ethmoid (superior)	0.846	0.002
Sphenoid (anterior)	0.706	0
Pakistani dataset		
Right maxillary	0.878	0.01
Sphenoid (anterior)	0.896	0.024
	0.757	0.001
Ethmoid (superior)	0.562	0
* Key results (EFA and linear measurements)*		
White British dataset		
Frontal	0.7	0
Ethmoid	0.672	0.001
Chinese dataset		
Frontal	0.469	0.002
Black Caribbean dataset		
Sphenoid	0.733	0
Indian dataset		
Ethmoid	0.74	0

**Table 4:** Cross-validated classification rates across age and sex

*Cross-validated classification rates*		
Sinus	Age% (*p* < 0.05)	Sex % (*p* < 0.05)
Chinese dataset		
Frontal	77.8	68.2
Sphenoid (anterior)	-	84.4
Sphenoid (superior)	84.6	-
Black African dataset		
Ethmoid (anterior)	71.7	
Ethmoid (anterior)	-	73.2
Indian dataset		
Ethmoid (superior)	50	-
Sphenoid (anterior)	41.2	73.5
Pakistani dataset		
Right maxillary	-	71.7
Sphenoid (anterior)	65.3	72.5
Ethmoid (superior)	79.6	-
* Cross-validated classification rates (linear and EFA)*		
Sinus	Age% (*p* < 0.05)	Sex % (*p* < 0.05)
White British dataset		
Frontal	-	76.6
Ethmoid	-	73.3
Chinese dataset		
Frontal	-	85
Black Caribbean dataset		
Sphenoid	-	72.6
Indian dataset		
Ethmoid	-	74.6

## Discussion

This study produced and evaluated 1500 three-dimensional reconstructions of the paranasal sinuses. Linear and elliptic Fourier analysis across six ethnic groups in a modern UK population was undertaken to determine if the size and shape of these structures can aid in establishing age, sex and ethnicity to unknown human remains and to identify which measurement variable/s are the most discriminant for use in crime reconstructions.

### To what extent can the size of paranasal sinuses discriminate on the basis of age, sex, and ethnicity?

The findings from this study demonstrate that the size of the paranasal sinuses can more reliably classify sex as opposed to age or ancestry. The results indicate that the size and shape of the paranasal sinuses showed a statistically significant relationship with age and sex, whereas predicting ethnic groups was not as successful as in other published studies [60, 61]. Although the results from the analysis of the entire sample demonstrated lower accuracy in classifying sex, these rates varied greatly within each ethnic group. For example, the Chinese dataset demonstrated that the right maxillary sinus provided an 85.7% classification rate in predicting sex. Additionally, the right maxillary of the Indian dataset showed an 80.4% (cross-validated at 69.6%) classification rate, and finally, the right maxillary within the Pakistani dataset did not show a statistically significant relationship according to sex.

The classification results from the consideration of the frontal sinus across the entire sample were not entirely consistent with previously published literature where size showed consistent patterns according to age [66–68] and did not show statistically significant relationships according to age in the frontal, maxillary or sphenoid sinuses. The Pakistani dataset was an exception, which provided a classification rate of 74.5% (cross-validated at 70.2%) for correctly classifying age in the left maxillary. Additionally, similar to previous literature, the volume was not a useful indicator to determine age [18]. This suggests, based on the results, that age-related patterns in the sinuses may discriminate between ethnic groups.

The classification results for predicting sex from frontal sinus measurements were similar to previous literature, where the classification rates from this study ranged from 62.7 to 75.0% [69, 70]. Hamed et al. [69] found a 67% accuracy in establishing sex, and Akhlagi et al. [70] showed classification rates ranging from 52.3 to 61.3% of the frontal sinus. However, the results from the frontal sinus Indian dataset showed a higher rate of sex classification (70.1% cross-validated at 64.2%) than previous studies that reported a 60% classification rate in estimating sex [71]. Finally, Michel et al. [18] employed 3D models of the frontal sinus to examine the frontal sinus volume in relation to sex and showed a classification rate of 72.5% which is in line with the classification rates demonstrated across the ethnic groups with the exception of the Pakistani dataset. The results indicated that differences in these results in sex classification may be the result of differences in data collection and visualisation methods where measurements taken from CT scans in comparison to 3D models may provide minor variations or may simply be due to differences between the populations under examination.

This variation suggests that this method is dependent on the population being considered which is crucial for understanding its employability for reconstruction purposes. Understanding the population-specific boundaries of this method is vital to encourage transparency in how well this method performs by specific populations rather than aiming to establish a ‘one-size-fits-all’ method.

The maxillary sinuses provided similarly inconsistent results across ethnic datasets in relation to sex classification. The datasets that demonstrated a statistically significant relationship to sex showed slightly lower classification rates than those recently reported [72–74]. The classifications documented in this study range from 57.8 to 85.7% with the left and right maxillary providing different results across the ethnic groups. The most promising results were found in Chinese and Indian datasets, where the classification rates ranged from 69.6 to 85.7%, where the right maxillary provided the highest classification rates. The results from this study are higher than other studies, showing 69% [75], 73% [76] or 70% [77] and 62% [78] classification rates for this sinus. This suggests that this method performed distinctly better than chance at correctly classifying sex, but also generally performed better than other published studies depending on ethnic group. However, the maxillary sinuses within the Black African and Pakistani datasets did not demonstrate a statistically significant relationship to sex, which was also displayed in Abasi et al. [79]. Furthermore, these results are in contrast with a range of literature where sexual dimorphism of these sinuses is demonstrated [72–78, 80–83]. This suggests that generalisations cannot be made with regard to sex estimations across ethnic groups within the maxillary sinus, but rather, it is dependent on the ethnic group under examination. These results also suggest that Black African and Pakistani datasets are not reliable for sex estimations for this sinus. Understanding these differences in variation between ethnic groups showed that particular results performed better than chance in classifying sex which could arguably be incredibly beneficial to future crime reconstructions.

Assessing whether the ethmoid sinuses could offer an indication of age and sex within a forensic context has not been attempted before. The ethmoid sinus only provided statistically significant classification results for the overall sample and within the Black African dataset in estimating age. For the overall sample, employing measurements from the ethmoid sinus provided a classification rate of 41.6% (cross-validated at 39.1%), with a classification rate of 65.8% (cross-validated at 60.5%) for the Black African dataset. Therefore, the documented classification rates presented here for predicting age groups may act as baseline results at this stage.

The ethmoid sinus over the entire sample and within the White British dataset did not show a statistically significant relationship according to sex. However, the other ethnic datasets did show a statistically significant relationship to sex with the Chinese, Black African and Black Caribbean datasets providing the highest classification rates, ranging between 63.0 and 76.3%. In contrast, Indian and Pakistani datasets showed lower classification rates in predicting sex, ranging from 61.9 to 67.9%. However, Paber et al. [84] showed a statistically significant difference in the height and the distribution of the Keros classification between Filipino males and females within the ethmoid sinus. The limited studies examining these structures in relation to sex make it difficult to place these results within the current literature which also highlights that this thesis is the first to demonstrate these trends within this sinus and therefore has provided baseline results.

The sphenoid did not show a relationship with age across the entire sample and across ethnic datasets. There are limited studies examining these structures in relation to age; however, the study by Yonetsu et al*.* [85] examined age-related expansion in the sphenoid sinus and showed that the volume of the sphenoid aeration started to decline and eventually decreased to two-thirds of its maximum level. The results demonstrated by Yonetsu et al. [85] and this study are therefore conflicting and may be due to differences between samples. The sphenoid sinus was the only sinus that showed a statistically significant relationship with sex within the Black Caribbean dataset. The classification rate of the sphenoid sinus within the Black Caribbean dataset was statistically significantly higher than the rates over the entire sample with a classification rate of 71.0% (cross-validated at 67.7%), while over the entire sample, the classification rate was 61.8% (cross-validated at 61.8%). The novelty in examining this sinus within this context means that there are limited studies available to compare within the current literature. Therefore, these results will also serve as a baseline for additional research to develop. However, in comparison with other sinuses, the sphenoid sinus did not show consistent results with or higher classification rates than the frontal or maxillary sinuses. This suggests from the results of this study that the sinus may not ultimately be useful in establishing age or sex overall; however, further research could usefully be undertaken to determine if sex classifications can be repeated within the Black Caribbean dataset.

### To what extent can the shape of paranasal sinuses discriminate on the basis of age, sex and ethnicity?

Paranasal sinus shape showed a statistically significant pattern between age and sex in varying degrees, with some cases showing that the ethmoid sinus provided the most discriminatory patterns. In contrast to the linear measurements, the EFA coefficients performed better in predicting age over the entire sample and within certain ethnic groups, such as Indian and Pakistani datasets. Across the paranasal sinuses, these patterns showed classification patterns according to sex, similar to the linear measurements. However, these results were not comparatively as high as the linear measurements, although these results varied according to ethnic group. For example, the White British dataset showed a classification rate of 67.5% for the EFA coefficients as opposed to the linear measurements of 70.5% (cross-validated at 68.2%). The Black African dataset showed similar results to the linear measurements. Finally, there were statistically significant relationships over the entire sample according to ethnic group, where classification rates ranged from 17.8 to 30.2%.

There is limited research available to place the results within the context of the current literature, but it is possible to compare these findings with studies that have addressed other anatomical regions. The classification rates demonstrated across these sinuses were generally lower than in other studies using geometric morphometrics on other skeletal regions [86–92]. Establishing age differences using geometric morphometrics has resulted in a prediction rate between 72 and 84% accuracy [92]. Millán et al. [91] also showed higher age-related changes using geometric morphometrics on the Os Coxae. These differences could be related to populational variations and differences in methodology and anatomical regions under study. However, the results from this thesis showed EFA coefficients performed more consistently at classifying age than the linear measurements.

The results from this study showed particular classification rates ranged between 73.2 and 84.4% for sex which are partially in line with current literature [89]. However, these results still did not perform as well as other studies [86, 88]. A majority of the dataset presented in this study showed lower classification rates ranging between 41.0 and 69.0% for sex. These results are also in line with several studies that did not find geometric morphometrics useful in establishing sexual dimorphism [93–96]. The extreme variations of the sinuses between individuals provide statistically significant limitations in employing the standard geometric analysis to assess sexual dimorphism, which limits the studies available for direct comparison. Cox et al. [97] used Elliptic Fourier coefficients to examine the shape of the frontal sinus and also did not find sexually dimorphic patterns. Overall, the paranasal sinuses did not demonstrate morphological differences to the same extent as other anatomical regions [87–91]. The varying classification ranges demonstrated are likely due to variations between the ethnic groups and are dependent on the skeletal region under examination. However, the results from the expanded sample size in this study indicate an increase in classification rates than initially identified in the preliminary study.

Over the entire sample, the maxillary, ethmoid and sphenoid sinuses demonstrated rates between 17.8 and 30.2% for classifying ethnic groups. These classification rates are in line with the classification rates reported for the linear measurements which are higher than chance (16.6%). Most of the coefficients in the preliminary study, although showing statistically significant group differences according to age or sex, did not provide a statistically significant discriminant model. This suggests that this method is extremely dependent on sample size and populations under examination. These results also indicate that the coefficients performed more consistently in predicting age and ethnic group than the linear measurements.

### Which variable/s are the most discriminatory predictor variable for establishing age, sex and ethnicity?

While both the linear measurements and EFA coefficients demonstrated their effectiveness in examining the relationship between age and sex as separate forms of measurement, testing these variables together was more successful in certain cases within specific ethnic groups. The results showed an increase in classifying age and sex across various ethnic groups but not over the entire sample. For example, within the White British dataset, the frontal sinus showed an increase in classifying sex, where volume and Siny 4 provided a 76.6% rate compared to the linear and EFA results separately. The ethmoid sinus showed an increase in the classification rate of 80.0% (cross-validated at 73.3%) for predicting sex, where the linear measurements only showed statistically significant ANOVA results and the EFA coefficients did not show a statistically significant relationship to sex. The results suggest that the combination of variables performed better in classifying sex than the linear or shape coefficients separately. However, there were still instances when certain linear and EFA results showed higher classifications when measured separately. For example, within the Black Caribbean dataset, the left maxillary showed a classification increase of 70.4% (cross-validated at 68.5%) in comparison to the linear measurements alone. However, the EFA coefficients tested alone still provided a higher initial classification rate at 75.9%. This suggests that, in this case, the EFA coefficients provided more discriminatory information regarding sex than the linear measurements and combination variables.

The results from this set of analyses did not demonstrate increases from the initial linear and EFA coefficients in classifying age or ethnic group across or within each ethnic group. However, the ability to classify sex using a combination of variables suggests that employing this method of analysis is more promising in finding sexual dimorphism than ageing or patterns in ethnicity in the paranasal sinuses. There are certain instances where the classification rates were higher than the linear measurements reported in other studies [69–71]. Furthermore, due to the limited studies examining the ethmoid and sphenoid sinuses, these results are the first to provide a baseline for the discriminatory ability of these sinuses using size and shape variables. Indeed, these results suggest that using a combination of variables of linear and EFA coefficients may be the most promising method to establish sex for unknown remains.

Finally, the ethmoid sinus has shown more discriminatory power than previously suggested, and it is worth investigating this structure along with the maxillary and frontal sinuses in further detail within a forensic anthropological context. Additionally, the sphenoid sinus did not provide statistically significant associations between age or ethnic groups but did demonstrate sexual dimorphism in the Black Caribbean dataset, which may prove useful for victim identification with further research. However, it is important to keep in mind while interpreting and comparing these results that the number of categories within the grouping variables (age/ethnic group) differs. This will influence the classification rates not only when comparing results within this study but also between existing studies that use different numbers of categories for classification. For example, a study that employs 2 age groups instead of 5 will show higher classification rates as the probability of allocation due to chance is increased (50% vs 20%). Similarly, within this study, comparisons of classification rates between age, sex and ethnic group must consider grouping differences. These grouping differences were noted in the text to make the interpretations clear. Indeed, this is a consideration when interpreting and comparing results across studies in forensic anthropology, where different sample sizes offer different boundaries and limits, thereby making direct comparisons less transparent. Arguably, the likelihood of a real-world scenario only involving two ethnic or age groups is limited, thereby emphasising the need for studies with a greater number of categories, such as the research resented here in this study. Finally, the classification rates presented in this study provide a baseline to improve upon, whereby this novel method may become a reliable supplementary source for establishing a biological profile of unknown skeletal remains.

### Emerging challenges and future research

The method presented in this study was designed to assist in DVI scenarios, where identification methods for a modern UK population that utilises imaging technologies are needed to assist in timely identifications [7]. Testing or developing new methods within the UK, especially when working from modern reference samples, offers huge benefits regarding representation and accessibility but also poses new problems to ancestry estimation [37, 39]. Discrepancies in definitions exist not only within forensic anthropology but also across wider academia and governmental institutions as well. For example, the UK government census page defines race as ‘a persons’ skin colour, nationality, and *ethnic* or national origins’ and defines ethnicity as ‘a shared history and culture, language, religion, and traditions, *as well as skin colour*’ [98]. This definition is substantially different from the definitions provided previously [38, 39, 99] and further highlights the numerous definitions and terms used across institutions [37].

This study differs from other forensic anthropology research because the known database that includes biological data, e.g. ethnicity is selected by the individual and not by the researcher, such as studies that classify ancestry or provide ancestral information that was decided by the institution/researcher (e.g. Hefner [100]; Hefner et al., [101]; Pilloud et al., [60]; Hefner et al., [102]). Therefore, this study had to not only confront the disparities between definitions of race, ancestry and ethnicity but also consider how governmental institutions and the public understand and interpret these terms within the UK. Therefore ‘self-identified’ NHS data and other similar terms are appropriate alternatives; however, they still contribute further tension to this discussion. Clarity behind the individual’s self-identified sex, age and ethnicity data may present new considerations with the final data interpretation and generalisability of the results.

The research presented here bridges interdisciplinary cooperation between medicine, forensic anthropology and technological innovation. Future developments of this method have the potential to utilise machine learning or artificial intelligence to improve the processing and data collection of CT images. Studies documenting improved workflow for processing CT images or automated sex estimation methods within forensic anthropology have recently been developed [3, 103, 104], thereby demonstrating an ongoing shift in future forensic anthropological research, where multi-disciplinary approaches should be employed and virtual methods that offer transparency and reproducibility are fully embraced.

## Conclusion

The aim of this research was to determine if the size and shape of 3D models of paranasal sinuses could assist in establishing the biological profile of unknown skeletal remains. The Robles et al*.* [6] method was followed to produce 3D models from CT scan data of paranasal sinuses, where the height, width, length and volume of the 3D models were first collected. Elliptic Fourier coefficients from the outline coordinates of the 3D models were then assessed, along with the linear measurements using discriminate function analysis, to establish how well these variables were able to predict age, sex and ethnic group. In summary:

- The *size* of the paranasal sinuses was a stronger indicator of sex than age or ethnicity.
- The results suggested that this method was ‘population specific’. In other words, higher accuracy rates in predicting sex were obtained when the ethnic group was known, as opposed to applied over the entire sample.
- The *shape* of the paranasal sinuses performed better and more consistently in classifying age, over the entire sample and within ethnic groups, in comparison to the size measurements.
- The shape of the sinuses performed better in classifying ethnic group over the entire sample in comparison to the size measurements.
- A combination of size and shape measurements of the paranasal sinuses reached higher classification rates according to sex within White British, Chinese, Black Caribbean and Indian datasets.
- A combination of size and shape measurements of the paranasal sinuses over the entire sample and within Black African and Pakistani datasets did not increase the classification rate for any sinuses according to age, sex or ethnic group.

These results were the first to demonstrate with a modern UK population that, in classifying age and sex, employing both size and shape measurements of the paranasal sinuses provided higher classification rates in certain instances than size and shape measurements separately. The application of employing both forms of measurements in the analyses was carried out to narrow down which sinuses and variables provided the highest predicted accuracy for establishing age and sex according to ethnic group for better practical application. This was demonstrated within White British, Chinese, Black Caribbean and Indian datasets which showed this method was the most successful within these datasets.

These findings indicate that the paranasal sinuses may be able to offer valuable discriminant characteristics that can be deployed in future forensic science settings. The fluctuating results presented are reflective of the variable nature of human beings, and while estimating consistent patterns across populations to establish a biological profile is complex, this study aimed to harness those variations into quantifiable patterns. The key development from this study was the use of an automatic approach to the 3D modelling method that facilitated and supported accurate data collection from a modern population. This underscores the potential capabilities of using computer-assisted methods based on 3D reconstructions of the sinuses to establish fast and reliable identifications. This study is the first of this scale within the forensic science context and begins to lay a foundation of new sources to assist with identifying patterns for biological profiles between populations, sex and age groups that have not been previously available.

### Supplementary Information

Below is the link to the electronic supplementary material.

Supplementary file1 (XLSX 295 KB)

## Data Availability

The CT scans and subsequent 3D datasets generated and analysed during the current study were derived from NHS patients and are therefore not publicly available due to ethical and legal restrictions.
